# Histopathological patterns and topographical distribution of Kaposi Sarcoma at Muhimbili National Hospital, Tanzania

**DOI:** 10.4314/ahs.v21i4.29

**Published:** 2021-12

**Authors:** Reginald Gervas, Edward Mgaya

**Affiliations:** School of Medicine, Muhimbili University of Health and Allied Sciences

**Keywords:** Kaposi's Sarcoma, Histopathological Patterns, Topographical distribution

## Abstract

**Background:**

Kaposi sarcoma (KS) is derived from endothelial cell lineage; it is caused by Human Herpes Virus-8 (HHV-8) facilitated by immune suppression. KS remains one of the commonest sarcoma seen in Tanzania. The paucity of recent data makes monitoring the disease a challenge. This study describes the Histopathological Patterns and Topographical distribution of Kaposi Sarcoma at Muhimbili National Hospital, a tertiary care hospital in Tanzania.

**Methods:**

A hospital-based retrospective cross-sectional study was done to review biopsies sent to the Central Pathology Laboratory (CPL), Muhimbili National Hospital from 2010 to 2014.

**Results:**

A total of 818 cases representing 1.8 % of all malignancies during the study period were enrolled in the study. The age of patients at diagnosis ranged from 6 months to 94 years old, with the median age being 37 years. Male to female ratio was 1.4:1.0. Females were younger than males (p < 0.001). The majority of the lesions were in the lower limbs, 352 (64.1 %). Nodular KS accounted for 74.5% of all cases.

**Conclusion:**

Kaposi's sarcoma remains a common malignancy. The patients present late at diagnosis. Early diagnosis and improved treatment protocols remain to be key steps towards reducing the burden of KS.

## Introduction

Kaposi sarcoma (KS) was first described by Moritz Kaposi, a Hungarian dermatologist in 1872; he described it as “idiopathic multiple pigmented sarcoma of the skin” 1. The etiological agent associated with KS was discovered in 1994 by Chang and his colleagues; it is called the Human Herpes Virus 8 (HHV-8), also known as KS-associated Human Herpes Virus 2. HHV-8 can be transmitted through saliva, organ transplantation and blood transfusion 3. The clinical relevance of KS includes both cosmetic disfigurement and considerable morbidity and mortality.

The epidemiological classification of Kaposi's sarcoma has four displays: the classic KS, the endemic or African KS, the iatrogenic KS and the epidemic (AIDS-associated) KS. Classic KS is a slow progressing neoplasm which affected mainly elderly men of Mediterranean and Eastern European region, especially Ashkenazi Jews 4. Endemic KS, also known as African KS was described in the 1950s. This form of KS has a more aggressive evolution than the classic form and the skin is extensively infiltrated; it affects both children and adults 5, 6. Iatrogenic KS, also known as Immunosuppression-associated KS develops in individuals such as those receiving organ transplant 7. The endemic KS, also known as AIDS-associated KS is a major AIDS-defining malignancy; usually, it develops among HIV patients who do not receive highly active antiretroviral therapy (HAART) 8.

Endemic (AIDS-associated) KS experienced an explosive growth in sub-Saharan Africa with the emergence of human immunodeficiency virus (HIV) infection in the region in early the 1990s 9. For example in Uganda KS became the leading cancer in males (about half of all registered cases) and the second most frequent (17.9%) in females in the early 1990s, this was the time when the AIDS epidemic was in its evolution in the region 10. The discovery and widespread use of highly active antiretroviral therapy (HAART) led to a tremendous decrease in the incidence of AIDS-related sarcoma (AIDS-KS) especally in developed countries^11^,^12^. The developing countries have been experiencing a modest decrease in the HAART era as compared to the developed countries^13^, ^14^. This may be attributed to late initiation of HAART (starting HAART when CD4+<200) before the test and start era, poor coverage of HAART, less use of more potent HAART such protease inhibitors in those failing treatment and other preventive or curative therapies for AIDS-associated cancers 15–17.

For unknown reasons, KS is more common in males than in females 13, 18. This male versus female incidence difference is more prevalent in HIV-negative adults 13. The clinical description of KS is the presence of hyper-pigmented lesions that grow in mucosa such as skin, mucous membranes lining the mouth, nose, throat or viscerally in lymph nodes, or other internal organs^19^, ^20^. The lower limbs are predominantly the most reported affected site by KS 13, 20, 21.

**Figure 1:**
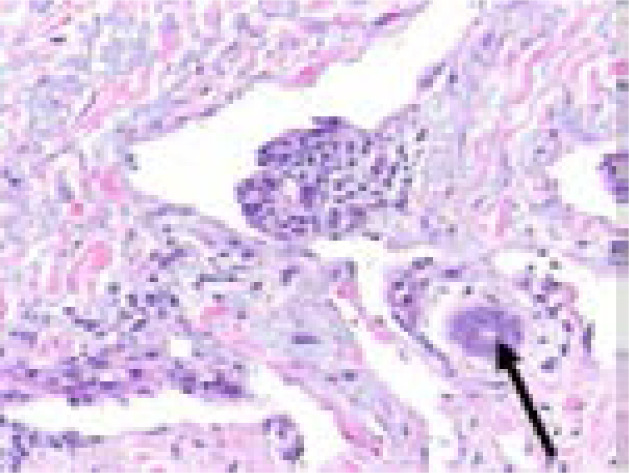
Patch stage Kaposi sarcoma showing newly formed vessels protruding into a larger vascular space characteristic of the promontory sign (H&E, 200X).

**Figure 2:**
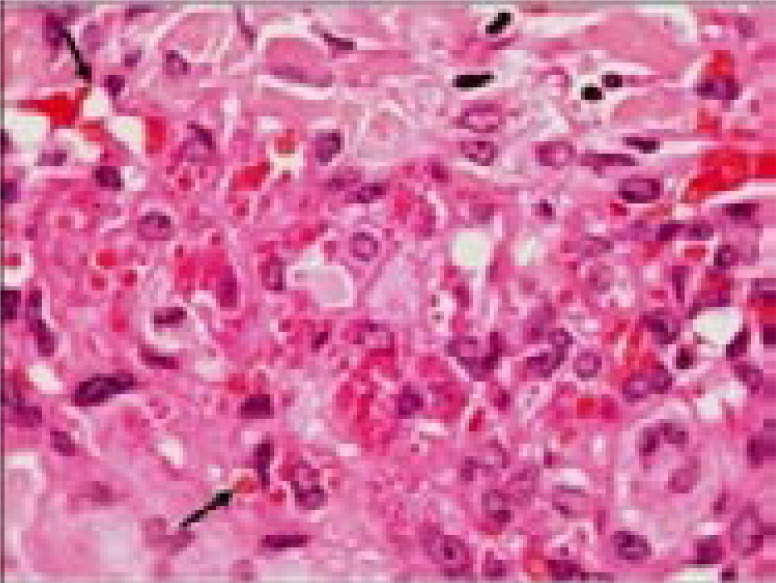
Plaque stage Kaposi sarcoma. numbers of intracellular and extracellular eosinophilic hyaline globules are visible in this field (H&E, 400X)

**Figure 3:**
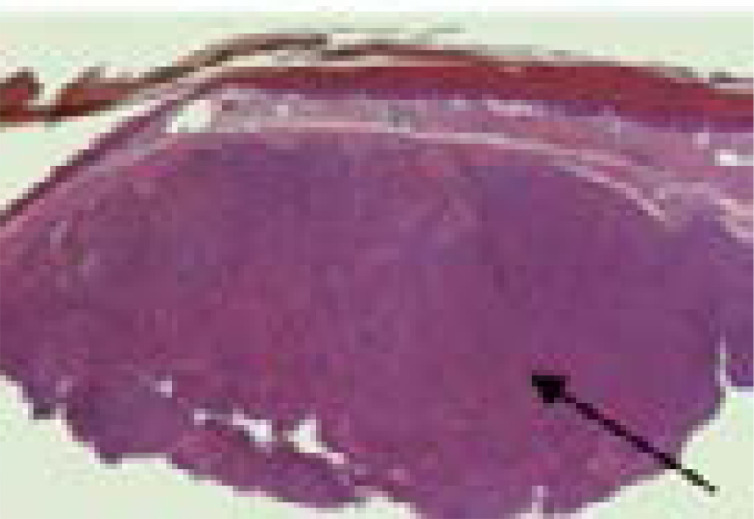
Nodular Kaposi sarcoma. The dermis is expanded by a solid tumour nodule (H&E, 20X).

The histology of KS lesions from the skin, lymph nodes and viscera is very similar. The histological hallmark of KS is the presence of spindle-shaped cells of endothelial origin with slit-like blood vessels and peripheral inflammatory cells 4. KS develops from patch (earliest focus of KS lesions), plaque (more indurated, oedematous and violaceous), or nodular (visible mass dominated by spindle cells) stages (Figure 1, Figure 2 & Figure 3) 22–24; the same patient can have lesions at different types of stages 6.

KS remains to be the leading malignant vascular tumour and second most prevalent cancer seen in Tanzania^15^, ^20^, ^25^. This necessitates closer monitoring on the KS in the country given that most patients seek medical care at late stages of the disease. However, despite this observation, there is a paucity of information regarding this condition in Tanzania. The aim of this study was therefore to describe the five-year experience in histological staging and topographical distribution of KS among patients receiving treatment at Muhimbili National Hospital, a national tertiary hospital in Tanzania.

## Materials and Methods

### Study site and data collection

This was a retrospective study whereby histologically confirmed cases of Kaposi's sarcoma from 1st January 2010 to 31st December 2014 were retrieved from the archives at Central Pathology Laboratory at Muhimbili National Hospital (MNH). MNH receives referred patients from all over the country, and it also serves as a national consultant hospital, a zonal referral hospital for East and Southern Tanzania as well as a university teaching hospital for Muhimbili University of Health and Allied Sciences (MUHAS). It has a 1,500-bed capacity, attending approximately 1,200 inpatients per week and approximately 1,200 outpatients per day.

Inclusion criteria were patients of both genders with histologically confirmed Kaposi's sarcoma.

Data concerning age, gender, histological type and topographic site of Kaposi's sarcoma were recorded. Patients' identifiers such as names were not recorded.

### Statistical data analysis

The statistical analysis was performed using the Statistical Package for Social Sciences (SPSS) version 20.0 for Windows (SPSS, Chicago, Illinois, USA). The student t test was used to test for significance of associations between the predictor and outcome variables in the continuous variables. The level of significance was considered as P < 0.05.

### Ethical approval

Ethical clearance was sought from the MUHAS Institutional Review Board and permission to conduct the study at the national hospital was granted by administration.

## Results

During the study period, a total of 45,250 malignancies were registered at Central Pathology Laboratory at MNH. Of these, 818(1.8 %) were histologically confirmed Kaposi's sarcoma giving an average of 164 cases annually. The age of patients at diagnosis ranged from 6 months to 94 years with a median of 37 years (IQR = 31–45 years). The modal age group was 31–40 years. Of all cases, 473 (57.8 %) were males and 345 (42.2 %) females; this gave male to female ratio of 1.4:1.0. Females were younger than males (mean age of 36.5 years vs. males 41.7 years; p < 0.001), Table 1.

**Table 1:** Age distribution

Age (years)	Male n (%)	Female n (%)	Total N (%)
<15	13 (2.8)	8 (2.3)	21 (2.6)
15–20	9 (1.9)	5 (1.5)	14 (1.7)
21–30	60 (12.7)	92 (26.9)	152 (18.7)
31–40	171 (36.3)	145 (42.4)	316 (38.9)
41–50	101 (21.4)	56 (16.4)	157 (19.3)
51–60	69 (14.6)	21 (6.1)	90 (11.1)
61–70	31 (6.6)	6 (1.8)	37 (4.6)
71–80	10 (2.1)	7 (2.0)	17 (2.1)
>80	7 (1.5)	2 (0.6)	9 (1.1)
**Total**	**473 (57.8)**	**345 (42.2)**	**818**

Table 2 shows the pathological stages and phases of KS. The lower limbs were the most common site for KS occurrence. In all cases, 352 (64.1 %) were present in the lower limbs. This was followed by the occurrence of KS in the oral cavity, 62 cases (11.3 %). There were two (2) cases (0.4 %) of KS in ovaries.

**Table 2:** Anatomical site of distribution of KS

Anatomical site	n (%)
Lower limb	352 (64.1)
Oral cavity	62 (11.3)
Upper limb	39 (7.1)
lymph nodes	24 (4.4)
Oropharynx	34 (6.2)
Head and neck	19 (3.5)
Trunk	7 (1.3)
Breast	4 (0.7)
Penis	4 (0.7)
Ovary	2 (0.4)
Bronchus	1 (0.2)
Anus	1 (0.2)
**Total**	**549**

* There were 167 biopsies from an unspecified part of the skin.

** There were 102 biopsies without a documented site of collection.

The most common pathological stage of KS observed in this study was the Nodular stage, this accounted for 461 cases (74.5 %) of all the KS lesions seen. Patchy and plaque stages accounted for the remaining 120 cases (19.4 %) and 38 cases (6.1%) respectively.

## Discussion

In this study, Kaposi's sarcoma accounted for 1.8 % of all malignancies recorded at the Central Pathology Laboratory in Muhimbili National Hospital in the 5-year study period from 2010 to 2014. This figure is lower than the figure of 2.4% reported Chayla et al in the Lake Zone of the country around the same time as this study 20. Another study at Ocean Road Cancer Institute (ORCI) deduced a decline in annual percentage of KS cases 10.1 % in 2003 to 7.4% in 2011; this was attributed to increased use of HAART during this period^25^. The reason for these high figures at ORCI might be because it is the only cancer centre in Tanzania; it offers both radiotherapy and chemotherapy.

Kaposi's sarcoma was more prevalent in males than in females, with a male to female ratio of 1.4:1.0. Male predominance has been observed in many other studies^21^,^25^–^27^. A study done at the same hospital 35 years ago revealed the male to female ratio to be 4.1:1 28. In South Africa, it is estimated that the incidence of KS has doubled in men while increasing by seven folds in women in the past 20 years 29. The difference in the rate of KS between the sexes reflects the emergence of AIDS-related KS which has reduced male to female ratio gap. A national survey that was conducted in Tanzania in 2016 revealed HIV prevalence in females to be as twice as much as compared to males, 6.2 % 30.

Kaposi's sarcoma has shown to affect all age groups and in this study, a 6 months old was diagnosed to have KS, the youngest ever patient diagnosed to have KS was 6 days old 31,32. On average, males were older than females. This is in agreement with other previous studies in sub-Saharan Africa 13, 21, 25, 29. Majority of the subjects fell between the ages of 31 and 40 years. This is the same age range that was most affected in the previous studies in sub-Saharan Africa 20, 33, 34.

In this study, the commonest sites of the disease were the skin, 77.6%. Skin presentation is the commonest mode of presentation, other sites especially the oral cavity and gastrointestinal tract are also common^25^, ^35^,^36^. This also might be attributed by lack of facilities and pro-activeness in the diagnosis of visceral KS in our settings as most of the patients might be mislabeled to have Pulmonary Tuberculosis or other chronic illness^27^. Overall, more than half of the patients presented with lesions in the lower half of the body (64 %). Lower limb KS predominance has been observed in many similar studies 7, 9, 13, 20, 25, 37. The reason for the lower limb to be the commonest site for Kaposi's sarcoma is not clearly understood.

Nodular KS accounted for 461(74.5%) of all KS biopsies in this study. Similar findings were seen in other studies in East Africa and the globe 33, 38, 39. Many studies have attributed this high prevalence of Nodular KS pattern to late presentation of the patients to healthcare facilities; many patients present in late stages for cancer diagnosis 20, 21, 25, 37.

## Conclusion

This study has shown Kaposi's sarcoma remains a common malignant tumour in the country. Majority of the patients presented with the disease in the fourth decade of their lives. The patients present late with the disease, they present with Nodular KS. Early diagnosis and improved treatment protocols remain to be key steps towards reducing the burden caused by this disease.
